# Exploring magneto-optic properties of colloidal two-dimensional copper-doped CdSe nanoplatelets

**DOI:** 10.1515/nanoph-2022-0503

**Published:** 2022-10-07

**Authors:** Avisek Dutta, Amani Saleh Almutairi, Jojo P. Joseph, Alexander Baev, Athos Petrou, Hao Zeng, Paras N. Prasad

**Affiliations:** Department of Chemistry and The Institute for Lasers, Photonics and Biophotonics, University at Buffalo, Suny, Buffalo, NY 14260, USA; Department of Physics, University at Buffalo, Suny, Buffalo, NY 14260, USA

**Keywords:** 2D, circular polarized photoluminescence, doping, magneto-optics, nanoplatelets

## Abstract

Transition-metal-doped semiconductor nanocrystals have received significant attention because of their attractive features deeming them invaluable in various technological fields including optoelectronics, bio-photonics, and energy conversion, to name a few. Of particular, these interests are two-dimensional materials with useful optical and magnetic properties combined with their large surface areas opening up new applications in biotechnology. These applications range from multimodal optical and magnetic bioimaging and sensing to measuring the weak magnetic field due to brain waves using their magneto-optic properties stemming from the exchange interaction between the transition metal dopants and the carrier spins. These magnetic 2D materials could also significantly advance the field of spintronics. In this work, we report on a study of the magnetic and magneto-optic properties of colloidal two-dimensional (2D) copper-doped CdSe nanoplatelets (NPLs) that are synthesized using a high-temperature colloidal technique. We carried out optical and circularly polarized magneto-photoluminescence spectrometry to investigate the magnetism in our solution-processed nanostructures doped with copper ion impurities. At cryogenic temperatures, two excitonic features are observed for doped NPLs, which are more prominent compared to the undoped NPLs. Furthermore, the excitonic circular polarization (CP) is recorded as a function of the applied magnetic field (*B*) and temperature (*T*). The detailed analysis provides a picture of the magneto-optical behavior of the doped 2D NPLs in the presence of paramagnetic copper ions. This work paves the way for significant advances in bio/nanophotonics where tunable optical and magnetic properties of doped nanoplatelets can be leveraged to make more efficient, flexible, and low-cost devices.

## 1 Introduction

Since their discovery a few decades ago, colloidal semiconductor nanoparticles with different shapes, morphologies, compositions, and dimensionalities have been demonstrated, such as zero-dimensional quantum dots (QDs) [1], one-dimensional quantum rods [2] or nanowires [3], and two-dimensional (2D) quantum disks [4] and nanoplatelets (NPLs) [5]. Among these different shapes and structures, two-dimensional nanostructures have emerged as of great interest [6–12]. In the 2D nanomaterials community, colloidal 2D NPLs have drawn a special interest because not only do they offer an ideal platform for a fundamental understanding of two-dimensional atomic crystals of traditional semiconductors but also offer potential advances in bio-nano-photonics, energy conversion, photodetection, and optoelectronics [13]. In particular, colloidal CdSe NPLs, exhibit some unique properties like narrow emission linewidth, high absorption cross-section, shorter lifetime, and high exciton binding energy compared to nanocrystals of different dimensionality [14, 15]. Therefore, efficient tuning of the optoelectronic properties of these NPLs has drawn significant attention. Changing the thickness of NPLs is a standard handle for tuning their optical properties [16]. Apart from changing the thickness, there are other ways to tune the properties of NPLs, of which doping has not been widely explored so far. Now, from the doping point of view, transition metal ion doping in QDs has been mostly explored for optoelectronic applications, owing to several advantages where manganese, cobalt, iron, copper, silver, etc. are generally used as the transition metal dopants [17–23]. Among all of the transition metal dopants, copper has several advantages over other dopants [24, 25]. Manganese doping can provide optical emission only at around 580 nm, which cannot be varied even with changing the host nanocrystal. On the other hand, copper doping can cover the whole range in the visible and NIR region depending on the host materials [26, 27] Moreover, copper-doped II–VI nanocrystals (NCs) are valuable optoelectronic materials with a wide range of potential applications [28]. From the mechanistic point of view, copper doping leads to an introduction of the inter-gap states between the conduction band (CB) and valence band (VB) of the host semiconductor, that which provides a useful tool for tuning the photoluminescence (PL) and the excited-state lifetime. Interestingly, copper can be doped as Cu^2+^ or Cu^1+^ in the host lattice, where, Cu^2+^ (3d^9^) has partially filled d-bands, and Cu^1+^ (3d^10^) has filled d-bands [29]. Consequently, the emission property is expected to strongly depend on the valence state of Cu in the NPLs. Studies on Cu-doped spherically shaped copper indium sulfide QDs revealed that Cu^2+^ based are emission-ready, while Cu^1+^-doped NPs require activation [30]. A few studies on the synthesis and characterization of Cu^1+^-doped NPLs have been reported earlier [31, 32]. Hence, it is increasingly important to synthesize Cu^2+^-doped CdSe NPLs and understand dopant-dependent photophysical and optical modifications. Despite the considerable effort to understand Cu- doped QDs systems, how the copper dopant emission behaves at lower temperatures, and their magnetic properties in the 2D phase remain unclear. Thus, a detailed analysis of the optical and magneto-optic data would be crucial for gaining further insight into photophysical processes in copper-doped NPLs systems. The magnetic properties acquired by semiconductor nanocrystals doped with Mn^2+^ ions with a half-filled d-shell have been explored relatively well [33]. Magnetically doped II–VI NCs, diluted magnetic semiconductor (DMS) systems, exhibit strong sp–d exchange interactions between the delocalized charge carriers of the host material (s-type electrons and p-type holes) and the localized magnetic moments of the dopants’ d-electrons. These exchange interactions lead to giant magneto-optic responses [34] and strong circularly polarized emission even at a small magnetic field [35], resulting in the observation of giant Zeeman splitting [36, 37]. In addition, it has been shown that spontaneous magnetization can be induced in Mn^2+^-doped CdSe NCs in the absence of an external magnetic field [33]. Copper-doped NPLs may show some interesting magneto-optic properties and, hence, deserve a detailed study.

Motivated by the above, we report here the synthesis and optical characterization of copper-doped CdSe NPLs. To investigate the magneto-optic properties of these NPLs we apply the magnetic circularly polarized luminescence (MCPL) technique. The effect of temperature and magnetic field on the doped NPLs has also been studied. We have compared the circular polarization in doped and undoped NPLs. It was found that in the doped NPLs, the circularly polarized PL is associated with the paramagnetic copper ions. This work provides insights into how the inclusion of paramagnetic dopants in NPLs can affect the magneto-optic properties.

## 2 Results and discussion

### 2.1 Morphological and optical characterization

We synthesized four monolayer-thick (4 ML) copper-doped and undoped CdSe NPLs using a high-temperature colloidal nucleation growth procedure. During the synthesis of CdSe NPLs, Cd (myristate)_2_, a long-chain carboxylate precursor, was dissolved in a long-chain organic octadecene solvent. Afterward, a short-chain ligand i.e., cadmium acetate [Cd (acetate)_2_], was introduced at a certain temperature when an anisotropic growth started from the seed to form 2D NPLs. For the doped NPLs synthesis, the copper stearate was injected into the solution flask before the addition of further cadmium precursor [Cd (acetate)_2_] to aid the appropriate doping of copper ions in the final doped CdSe NPLs. Furthermore, to get the final product for heavily doped NPLs, the as-synthesized doped NPLs were treated with some thermal annealing.

**Figure 1:**
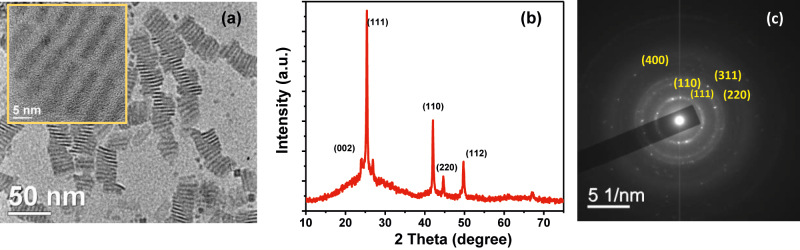
Structural characterization of Cu-doped NPLS; (a) TEM image of Cu-doped CdSe NPLs (inset: HRTEM image), (b) XRD pattern, and (c) SAED image of Cu-doped CdSe NPLs.

After the synthesis, we carried out a detailed analysis of the synthesized doped and undoped NPLs with transmission electron microscopy (TEM) and X-ray diffraction study (XRD) for structural characterizations. Figure 1a shows the TEM image of copper doped CdSe NPLs and the inset picture shows the high-resolution TEM (HRTEM) image of the doped NPLs. The lateral area of the NPLs was found around 210 ± 40 nm^2^. Figure 1b shows the XRD pattern of Cu-doped CdSe NPLs. XRD peaks are not broadened uniformly which might be due to a highly anisotropic 2D morphology and layer stacking disorder. The relatively narrow line width of the peaks in XRD represents the corresponding planes of the doped sample and that may be ascribed to the anisotropic growth of the crystallites. It results in larger lateral dimensions for the doped samples than for the undoped sample (Figure S1). The zinc blende (ZB) phase is primarily responsible for providing the nanoplatelets structure of the doped NPLs in XRD [38]. But, the wurtzite phase might coexist with the major ZB phase in the crystal lattice for the doped NPLs [38]. However, as shown in Figure S1, the XRD patterns of undoped CdSe NPLs show the most intense (111) peak at 25.3° 2*θ*. In the case of highly anisotropic 2D CdSe NPLs, all these peaks severely overlap and form a broad asymmetric peak. Figures 1c and S1 show the selected area electron diffraction (SAED) images of the doped and undoped NPLs, respectively, where all planes mentioned in the XRD pattern are clearly visible. The rings in the SAED are highly intense and broad which reveals a predominance of the characteristic patterns of crystalline ZB CdSe structure. Four prominent rings are observed which correspond to (111), (220), (311), and (400) planes, respectively, and it matches the previously discussed XRD planes on doped NPLs. Atomic force microscopy (AFM) images of undoped and doped NPLs show a similar appearance to NPLs where the height profile along slightly stacked areas (Figure S2) shows a thickness of almost 1.4 ± 0.2 nm. The successful incorporation of copper ions in CdSe NPLs was confirmed from the inductively coupled plasma optical emission spectroscopy (ICP-OES) analysis for the cadmium, selenium, and copper in the doped NPLs where we have found around 3% copper, which corroborates stoichiometry used in the synthesis. We can explicitly observe the presence of copper ions along with Cd and Se elements. Electron paramagnetic resonance (EPR) study confirms the paramagnetic feature which is due to the presence of some portion of the Cu^2+^ (d^9^) (Figure S3).

**Figure 2:**
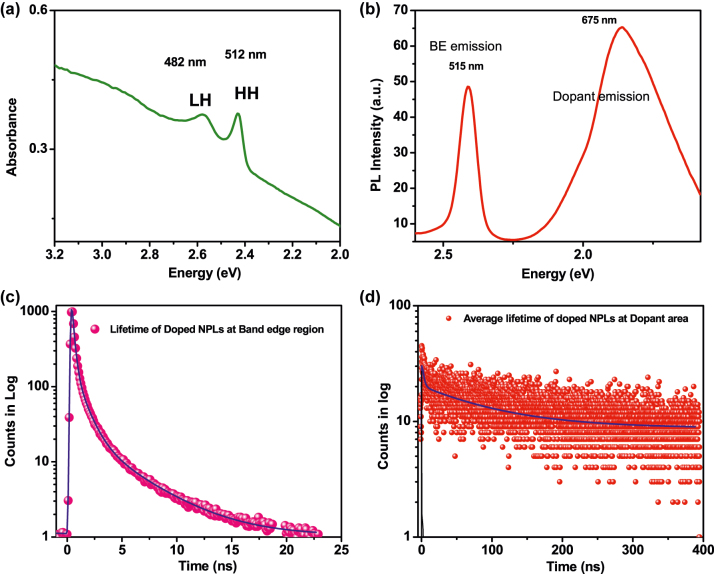
Optical characterization of the doped NPLs; (a) UV-vis and (b) photoluminescence (PL) spectrum of Cu-doped CdSe NPLs, (c) average decay profile of doped NPLs at band edge emission area, and (d) average decay profile at dopant emission area.

For optical characterization, we performed the steady-state UV-visible absorption and photoluminescence (PL) spectroscopic studies on undoped, and copper doped CdSe NPLs. Steady-state UV-vis and PL spectra of undoped CdSe NPLs show absorption at 2.43 eV (509 nm) and 2.58 eV (480 nm) and a sharp emission at 2.42 eV (511 nm) having a full width at half maximum (FWHM) of ∼10 nm with almost no Stokes’s shift (Figure S4). The UV–vis absorption peaks for the doped NPLs have appeared at 2.42 eV (512 nm) and 2.57 eV (482 nm) (Figure 2a), corresponding to the excitonic transitions of the heavy hole-electron and light hole–electron pair. Copper doped CdSe NPLs exhibited a broad characteristic PL emission at around 1.84 eV (675 nm) in addition to the band-edge emission at 2.40 eV (515 nm) (Figure 2b). This broad lower energy emission band has a large Stokes shift relative to the band edge of the host and a greater FWHM than undoped NPLs. A similar large Stokes shifted dual emission band has been observed in Cu-doped II–VI quantum dots system in an ensemble [39, 40] and in the single molecular state of Cu^2+^-doped CdSe NCs case [41]. It has been also discussed recently that the band-edge optical properties of CuInS_2_ QDs samples are strongly influenced by native optically active copper defects [42]. It has been reported that the Cu^2+^ (3d^9^) oxidation state introduces an optically active intragap hole above the Fermi level of the semiconductor host matrix [39]. In the case of the d^9^ system, there are two channels for relaxing the photoexcited electrons from the conduction band to the valence band. The first channel comprises the band edge emission due to the relaxation of the conduction band electrons with valence band holes via radiative recombination. The second channel comprises dopant emission arising due to the radiative decay of photoexcited electrons to the optically active Cu^2+^ dopant hole. In contrast, Cu^1+^ (3d^10^) doping introduces an intragap d-state below the Fermi level and the dopant emission is observed only when the valence band hole transfers to the intragap d-states, as evident in Cu^1+^ doped CdSe NPLs. Furthermore, the photoluminescence excitation spectrum of the Cu-doped CdSe NPLs (Figure S5) was collected at the 675 nm dopant emission wavelength, which confirms that the doped PL emission comes from the nanoplatelets. The PL excitation spectrum resembles well the UV-vis absorption spectra. These data from the optical characterization confirm that copper dopant emission arises from the recombination of conduction band electrons and holes in copper levels, which supports successful copper doping in NPLs.

The time-resolved PL decay measurements were carried out on undoped, and Cu-doped CdSe NPLs using the Leica TCS-SP8 confocal instrument. We measured the average lifetime of the photoexcited carrier in the band edge and dopant emission range. The decay profiles do not show a monoexponentially decay, which is common for colloidal nanocrystals. The decays are fitted with a three-terms exponential function. As a result, the band edge carrier average lifetime of Cu-doped CdSe NPLs is found to be 0.9 ± 0.2 ns (Figure 2c), whereas the band edge lifetime for undoped samples is found to be 1.1 ± 0.3 ns (Figure S6).

Interestingly, the dopant lifetime of >100 ns is much higher compared to the band edge lifetime (Figure 2d). The significant increase in the average lifetime compared to the band edge due to the weak spatial overlap between the conduction band electron wave function and the localized copper level further confirmed the successful incorporation of the copper dopant ion which was also observed for the doped quantum dots system.

**Figure 3:**
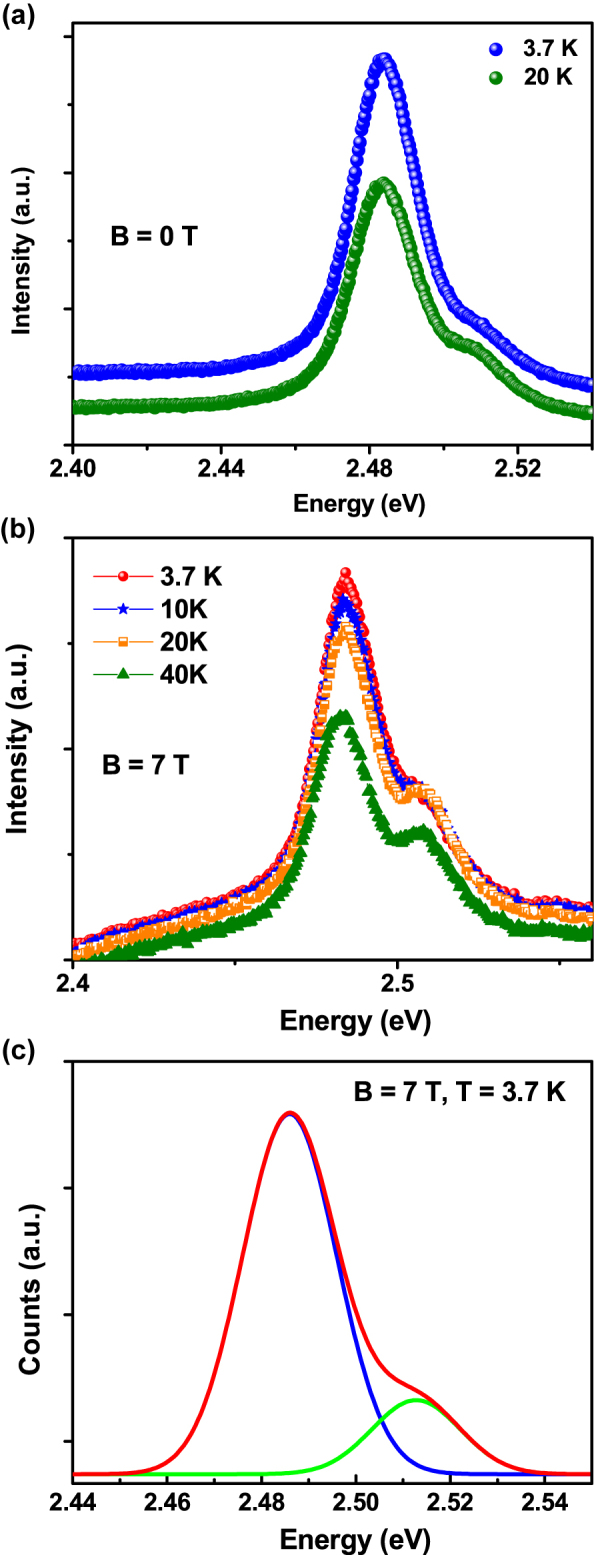
Temperature effect on PL properties with and without magnetic field. (a) Low-temperature PL study of Cu-doped CdSe NPLs at different lower temperatures without magnetic field (*B* = 0 T). (b) Temperature dependence of excitonic features at 3.7 K (red), 10 K (blue), 20 K (orange), and 40 (green) with applying magnetic field (*B* = 7 T), (c) the magneto-PL fitting in the vicinity of excitonic emissions. Bands in green and blue are recognized as free-exciton and bound-exciton emissions, respectively.

Next, we investigated the effect of lowering the temperature and applying an external magnetic field on the band-edge luminescence. Sometimes, it is hard to get the MCPL and CP values due to the weakness of the band edge excitonic emission like in Ag^1+^-doped nanocrystals [43]. Thanks to paramagnetic copper dopant ion, we observed band edge PL along with dopant PL which helps to investigate the *para*-magnetization properties. Figure 3a shows the PL emission of the doped NPLs at low temperatures without applying any magnetic field. It is noted that we observed maximum PL intensity at a lower temperature when we compared the PL emission data at 20 and 3.7 K (Figure 3a). We observed a small splitting in the excitonic PL at 2.51 and 2.48 eV, respectively. Interestingly, after applying a magnetic field, the splitting of the band edge excitonic PL becomes more prominent. Band edge PL spectrum in Figure 3b exhibits two clear features at high and low energy. They are identified as the free and bound exciton respectively. Temperature dependence of excitonic features at 3.7 K, 10 K, 20 K, and 40 with applying a magnetic field (*B* = 7 T) is shown in Figure 3b. Based on the dependence of their relative intensities on *T*, we have found that from 3.7 to 40 K the intensity of the fee exciton relative to the bound exciton increases. In Figure 3c, we show a fit of the data shown in Figure 3b at a lower temperature and at a 7 T magnetic field. Here, the band-edge luminescence is represented by the red-colored line which is fitted with two Gaussian line shapes: Feature (a) (green) at 2.52 eV and feature (b) (blue) at 2.49 eV are identified as being due to the free exciton and bound exciton, respectively. The free exciton is a mobile electron−hole hydrogenic system, while the bound exciton is a complex hydrogenic system localized on an impurity atom. The little broadening of the free and bound excitonic features when compared to the room temperature PL spectrum could be attributed to the interaction with residual impurities for dopant ion insertion or any kind of strain generation after doping within the nanoplatelets or the effect of temperature. At low temperatures, the PL contains features associated with both the free and the bound exciton. At room temperature, the excitons that were bound become free, and the PL contains only a free excitonic feature.

**Figure 4:**
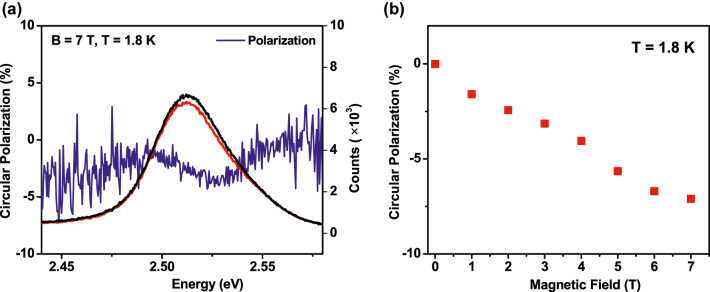
Circular polarization with the effect of magnetic field. (a) Right circularly polarized (RCP) (black) and left circularly polarized (LCP) (red) components of PL of the Cu-doped sample at *T* = 1.8 K and *B* = 7 T. (b) Circular polarization of the exciton as a function of magnetic field (B) at a particular temperature (*T* = 1.8 K) for the Cu-doped CdSe NPLs sample.

To investigate the influence of magnetic copper ion doping, we conducted the MCPL spectroscopy and measured the band edge CP as a function of magnetic field (*B*) at *T* = 1.8 K. The results are summarized in Figure 4. The magnetic field was applied in the Faraday geometry, which is parallel to the PL emission wave vector direction. At 1.8 K, left circularly polarized; LCP (σ+) and right circularly polarized; RCP (σ−) components of band-edge PL have different intensities and that results in non-zero circular polarization (blue line). Figure 4a shows the polarization at *B* = 7 T as a function of photon energy for the doped NPLs at 1.8 K temperature. The circular polarization (CP) is defined as 100 × [I (LCP) − I (RCP)]/[(I (LCP) + I (RCP)], where I (LCP) and I (RCP) are the intensities of the LCP (σ+) and RCP (σ−) PL components, respectively. The zero-field CP has a value close to +3%. Its origin is unknown but spontaneous polarization has been observed in 2D transition metal dichalcogenides and other semiconductor quantum dot systems [44]. But, we attribute the non-zero polarization at *B* = 0 in both samples to the strain in the optical cryostat windows during cooling. Hence, we have subtracted the zero-field positive value of circular polarization at *B* = 0 from the data obtained at different values of the magnetic fields. With the application of an external magnetic field, the CP acquires a systematic negative value at the energy of the free exciton, as indicated in Figure 4b. At 7 T, we observed a maximum CP value of 7%. In the case of the undoped NPLs, the value of CP is non-zero (∼1%) at *B* = 0 T and a similar subtraction of the positive value of circular polarization at *B* = 0 has also been used for this sample. After applying the magnetic field, the CP value changes in the case of the undoped NPLs as shown in Figure S7, with the obtained CP value of −1% which is significantly smaller than the −7% of the doped sample. The excitonic circular polarization is plotted as a function of the magnetic field, B, for the undoped and doped samples in Figures S8 and 4b, respectively. We noticed that the field dependence of CP on B is linear and has a negative slope in the case of undoped NPLs. For the doped sample, the CP retains the negative slope and obtained a higher polarization value with increasing the magnetic field with compared to the undoped case. By comparing the data of 6 and 7 T one can get a hint of the saturation of the polarization at a higher magnetic field because the slope is getting saturated. This saturation might be more prominent at a higher magnetic field. For example, in the Mn^2+^-doped QDs system the saturation has been observed at 15 T [45]. In contrast with the copper-doped sample, the CP of the undoped sample does not exhibit any sign of saturation as shown in Figure S8.

The magnetic field versus magnetic moment plot shown for copper-doped CdSe NPLs in Figure S9 shows vibrating-sample magnetometer (VSM) results, where we observed that the magnetic moment increases linearly with increasing magnetic field, which is typical for a paramagnetic material. We interpret that at least a fraction of copper is present as Cu^2+^ with a non-zero magnetic moment and as an outcome, we have got the appearance of paramagnetism in our doped sample. During the study of MCPL, after photoexcitation, electrons are photoexcited into the conduction band and then recombine either with the holes in the CdSe valence band as an excitonic channel as well as a hole created in the copper d-state. To ensure the emission for Cu^2+^ we irradiated our sample for a few minutes before measurement to increase the population of Cu^2+^ paramagnetic ions if some Cu^1+^ ions were still present. Arman et al. have used a similar technique where Ag^1+^ was converted into Ag^2+^ after illumination [46]. They observed that after photoexcitation, some of the electrons in the conduction band can get trapped at the surface defect states. As a result, if a majority of photogenerated holes are created in the valence band, a number of these holes could be captured by the nonmagnetic Ag^1+^ 4d^10^ state and it could result in the magnetic 4d^9^ Ag^2+^ state. In our case, Cu^2+^ paramagnetic ions are already present in the as-synthesized samples as discussed previously, which leads to sp−d exchange interactions responsible for the observed enhanced polarization. It has been reported that the polarization is positive in the case of Mn-doped Quantum dots [47]. A sign reversal in the polarization i.e. a negative polarization was observed for the Ag^1+^ doped or doped ZnSe–CdSe NCs [46, 48]. In our system, we also observed a negative polarization for Cu-doped NPLs, which was attributed to the nature of the interaction between holes in the valence band and unpaired electrons in the dopant d state that mediates the magnetic behaviour of the doped NPLs.

## 3 Conclusions

In conclusion, a high-temperature colloidal synthesis of 2D CdSe NPLs doped with paramagnetic copper has been described. The morphological and optical properties were studied, confirming the successful doping of copper ions into 2D NPLs. We observed a sharp excitonic PL feature at the band edge and a broad PL at lower energy associated with the dopant which has a longer average lifetime compared to that of the band edge lifetime. Low temperature PL measurement and MCPL spectroscopic studies have been conducted to investigate the excitonic optical manipulation of magnetism in our copper-doped CdSe NPLs. We measured the circular polarization as a function of the magnetic field, B, and temperature, T, and recorded a decrease in CP with increasing the B. The enhanced magneto-optic response in copper-doped NPLs compared to the undoped CdSe NPLs is attributed to sp–d interaction. Hence, this work provides insights into how doping of paramagnetic ions into NPLs can affect their magneto-optic properties. This system can be considered a promising magneto-resistive biomedical platform, as well as a solid foundation for the 2D solution-processed spin-based semiconductor devices and other spintronic applications.

## 4 Experimental methods

### 4.1 Materials

Myristic acid, cadmium nitrate tetrahydrate [Cd (NO_3_)_2_·4H_2_O], sodium hydroxide (NaOH), selenium powder, 1-octadecene (ODE, 90%), oleic acid (OlAc) (90%), cadmium acetate dihydrate [Cd (OAc)_2_·2H_2_O], stearic acid, and copper (II) nitrate [Cu (NO_3_)_2_] were purchased from Sigma Aldrich. Solvents like acetone, ethanol, methanol, toluene, and hexane were obtained from Decon laboratories INC, Avantor, and sigma Aldrich.

### 4.2 Material synthesis

#### 4.2.1 Preparation of cadmium myristate

Cadmium myristate [Cd (Myr)_2_] was synthesized by following previously published procedures with some modifications. To prepare cadmium myristate, a sodium myristate solution was prepared by dissolving 0.43 g of sodium hydroxide and 3.42 g of myristic acid in anhydrous methanol (200 mL). In another beaker, 1.11 g of cadmium nitrate tetrahydrate was dissolved in 40 mL methanol. The cadmium nitrate solution was added dropwise into the sodium myristate solution with stirring at room temperature. After that, a white precipitate of cadmium myristate starts to appear. When the addition of cadmium nitrate solution was completed, the reaction was allowed to stir for 2 h more. Finally, the precipitate was washed with dried methanol several times to remove the unreacted precursors, followed by drying at 60 °C under a vacuum for a few hours.

#### 4.2.2 Preparation of copper stearate

Copper stearate was synthesized using the previously reported method with some modifications [29]. Stearic acid of weight 2.84 g was dissolved in 20 mL of methanol in a conical flask and heated to 50 ^o^C until a clear solution was observed. TMAH of weight 2.93 g was dissolved in 5 mL methanol separately and added to the stearic acid solution dropwise. The mixture was vigorously stirred for 20 min 1.2 g of Cu (NO_3_)_2_ was dissolved in a separate conical in 10 mL methanol and added dropwise to the above solution under continuous stirring conditions. Sky blue precipitates of CuSt_2_ were collected by centrifugation and washed thoroughly with methanol and acetone. Finally, copper (II) stearate was dried overnight under a vacuum.

#### 4.2.3 Synthesis of colloidal undoped 4 ML CdSe NPLs

4 ML CdSe NPLs were synthesized by solution-phase colloidal synthesis with some modifications [49]. Briefly, in a three-neck flask, 85 mg of Cd (Myr)_2_, 6 mg of Se powder, and 15 mL of ODE were degassed at 90 °C for 1 h. Then, the flask was put into an argon atmosphere and heated to 240 °C rapidly under continuous argon flow. When the color of the solution became yellowish-orange at 195 °C, 20 mg of Cd (OAc)_2_ was added immediately. The solution was kept at 240 °C for 10 min, and then the reaction was stopped. Oleic acid of 1 mL was added during the reduction of temperature. Finally, the mixture was dissolved in hexane, and by size-selective precipitation, the byproducts like quantum dots were excluded from the mixture. The precipitate contained undoped 4 ML CdSe NPLs which were re-suspended in toluene for further uses.

#### 4.2.4 Synthesis of copper-doped CdSe NPLs

Copper-doped 4 ML CdSe NPLs were synthesized by solution-phase colloidal synthesis procedure. Briefly, 85 mg of Cd(Myr)_2_ (0.3 mmol), 6 mg of Se powder, and 7.5 mL of ODE were taken in a two-neck round bottom flask and degassed at 90 °C for 1 h. Then, the temperature was increased rapidly to 240 °C with continuous stirring under an argon atmosphere. At 190 °C, when the solution turns straw yellow color, previously synthesized solid copper stearate (3 mol % with respect to total Cd^2+^) was added to the reaction mixture. 20 mg of Cd (OAc)_2_ was added at 195 °C. The solution was kept at 240 °C for 10 min. Then, the reaction was stopped and cooled immediately. When the temperature reaches 60 °C during cooling, 1 mL of oleic acid was added. The reaction mixture was dissolved in hexane and centrifuged at 6500 rpm for 7 min. The supernatant containing quantum dots and other impurities was extruded. Finally, the precipitate was washed using a mixture of hexane and ethanol. The final Cu-doped 4 ML CdSe NPLs was collected after the centrifugation at 5000 rpm for 8 min. The doped NPLs were dried under a vacuum and suspended later in toluene and kept as a final after post-synthetic thermal annealing treatment for 24 h for further morphological, optical, and magneto-optical characterizations.

### 4.3 Characterizations

Powder X-ray diffraction (XRD) was recorded using a Rigaku Ultima IV XRD powder diffractometer. Transmission electron microscopy (TEM) measurements were done by using a JEOL, JEM-2010, at an operating voltage of 200 kV. TEM samples were prepared by drop-casting of NPLs solution in toluene on a carbon-coated copper grid followed by the evaporation of the solvent. Electron paramagnetic resonance (EPR) spectrum was measured in microwave frequency on a Bruker Bio Spin Corporation. The measurement was recorded at a lower temperature with liquid nitrogen. Inductively coupled plasma optical emission spectroscopy (ICP-OES) was performed using Thermo Scientific iCAP 6000 instrument. Samples were prepared several times washing and dried after that. Washed NPLs were then digested in concentrated HNO_3_ and diluted with Millipore water. Room-temperature optical UV-visible absorption spectra were recorded by a UV-vis NIR scanning spectrophotometer (Shimadzu). Steady-state PL studies were carried out by using a Shimadzu Spectro fluorophotometer (RF – 5301 PC). The average lifetime was measured with Leica TCS-SP8 confocal instrument. The repetition rates were changed depending upon the lifetime range of the samples. For magneto-PL measurements, the samples were placed in a closed cycle refrigerator (CCR) for zero fields PL and transmission work. A closed cycle of 7 T optical magnet cryostat was used. Experiments were conducted in Faraday geometry where the applied magnetic field is parallel to emitted PL. The PL spectra were excited using the linearly polarized output of a 405 nm diode laser. The emitted PL was collected and analyzed by a single monochromator equipped with a cooled CCD multichannel detector. The LCP (σ+) and right circularly polarized RCP (σ−) components of PL were separated using a combination of a quarter wave plate and a linear analyzer placed just before the spectrometer entrance slit.

## Supplementary Material

Supplementary Material Details
