# Supplementary material for: Exploring magneto-optic properties of colloidal two-dimensional copper-doped CdSe nanoplatelets
Source: Nanophotonics. 2022 Oct 7;11(22):5143–52. doi: 10.1515/nanoph-2022-0503 (PMC11501793; doi:10.1515/nanoph-2022-0503)
Supplement: Supplementary file 1 — Supplementary Material Details [file j_nanoph-2022-0503_suppl.docx]

**Supporting Information (SI)**

**Exploring Magneto-Optic Properties of Colloidal Two-Dimensional Copper Doped CdSe Nanoplatelets**

Avisek Dutta,^1^ Amani Saleh Almutairi,^2^ Jojo P. Joseph,^1^ Alexander Baev,^1^ Athos Petrou,^2^ Hao Zeng,^2*^ and Paras N. Prasad^1,2*^

*^1^ Department of Chemistry and The Institute for Lasers, Photonics and Biophotonics,*

*University at Buffalo, SUNY, Buffalo, New York 14260, United States*

*^2^Department of Physics,*

*University at Buffalo, SUNY, Buffalo, New York 14260, United States*

**Figure S1:** (a) TEM images of undoped CdSe NPLs (inset: HRTEM image), (b) the SAED image of undoped CdSe NPLs, and (c) XRD data of undoped CdSe NPLs

**Figure S2:** AFM images of undoped NPLs (upper) and doped NPLs (lower).

**Figure S3:** EPR spectra of Copper doped CdSe NPLs

**Figure S4:** UV – Vis and PL spectra of undoped CdSe NPLs.

**Figure S5:** Excitation spectra of Copper doped CdSe NPLs at dopant emission wavelength.

**Figure S6:** Average decay profile of undoped CdSe NPLs measured at band edge area.

**Figure S7:** Magneto-PL of undoped CdSe NPLs; (a) at 0 T and (b) 7 T at a particular cryogenic temperature (3.7 K).

**Figure S8:** Circular Polarization as a function of magnetic field (B) at a lower temperature (3.7 K) for the Cu doped CdSe NPLs sample.

**Figure S9:** Magnetic Field vs Magnetic Moment plot for copper doped CdSe NPLs.
